# Celiac Artery Aneurysm: A Rare Cause of Abdominal Pain

**DOI:** 10.7759/cureus.48494

**Published:** 2023-11-08

**Authors:** Duwayne Campbell, Wayne Tamaska, Sergey Medlenov, James Espinosa, Alan Lucerna

**Affiliations:** 1 Emergency Medicine, Jefferson Health New Jersey, Stratford, USA

**Keywords:** emergency medicine and visceral aneurysms, emergency medicine management of celiac artery aneurysms, abdominal pain due to a celiac artery aneurysm, visceral artery aneurysm, celiac artery aneurysm

## Abstract

We present the case of a 48-year-old male who presented to the emergency department with left-sided abdominal pain of four-day duration. The pain was described as sharp in nature and was located in the left lower quadrant with radiation to the left shoulder.

A computerized tomography (CT) scan of the abdomen and pelvis without contrast showed celiac artery enlargement with adjacent inflammatory stranding. There were other chronic findings secondary to prior surgery. A CT angiogram of the abdomen and pelvis was performed, which showed a 10 x 5 mm saccular pseudoaneurysm at the posterior aspect of the celiac trunk with surrounding inflammatory changes. Endovascular repair was accomplished in the operating room after the placement of the stent graft. Repeat angiogram showed successful exclusion of the aneurysm, with excellent perfusion to the arteries distal to the site of repair. The patient was successfully discharged two days later with outpatient follow-up. Celiac artery aneurysms can present to the emergency department with abdominal pain. The detection of celiac artery aneurysms may be increasing due to increased detection on CT scans. Although rare, this type of visceral artery aneurysm carries a high mortality rate if ruptured. Surgical repair may be either through an endovascular approach or through open surgical repair of the aneurysm preferably with prosthetic grafts.

## Introduction

A visceral artery aneurysm (VAA) can involve the celiac artery, the superior mesenteric artery, the inferior mesenteric artery, and the branches of these arteries [1]. Celiac artery aneurysms (CAAs) are rare, and account for only 4% to 5% of all VAAs [2]. However, a CAA can be accompanied by another aneurysm, such as an abdominal aortic aneurysm (9% accompaniment rate) [2]. Rupture occurs in 10-20% of CAAs [3]. The mortality rate with rupture has been noted to be 50% [3]. When a rupture occurs, it usually occurs into the peritoneum or the retroperitoneum [4].

This case report was previously presented as a poster as part of the Rowan University Research Day, on May 5, 2022.

## Case presentation

A 48-year-old male presented with left-sided abdominal pain of a four-day duration. The pain was described as sharp in nature and was located in the left lower quadrant with radiation to the left shoulder. There were no associated symptoms. He denied any aggravating or relieving factor to the pain. The patient was status-post laparotomy, splenectomy, partial pancreatectomy, with bowel resection and colostomy with colostomy reversal all from a gunshot wound to the abdomen 12 years prior. He had no other significant past medical history and was not taking any medications. The patient's vital signs revealed a heart rate of 110 beats per minute. The remaining vital signs were as follows: respiratory rate of 18 breaths per minute, blood pressure of 136/82 mmHg, and temperature of 98.8 degrees Fahrenheit with an oxygen saturation of 99%. On physical exam, he was noted to have left lower quadrant tenderness, with no peritoneal signs. The physical exam was otherwise unremarkable.

Laboratory tests were ordered, including a complete blood count, basic metabolic panel, lactate, lipase, urinalysis, and blood cultures. The laboratory test results showed a white cell count of 10,700 per mm^3^ with 82% neutrophils and a CRP of 1.0 mg/L. The laboratory results were otherwise within normal limits.

**Figure 1:**
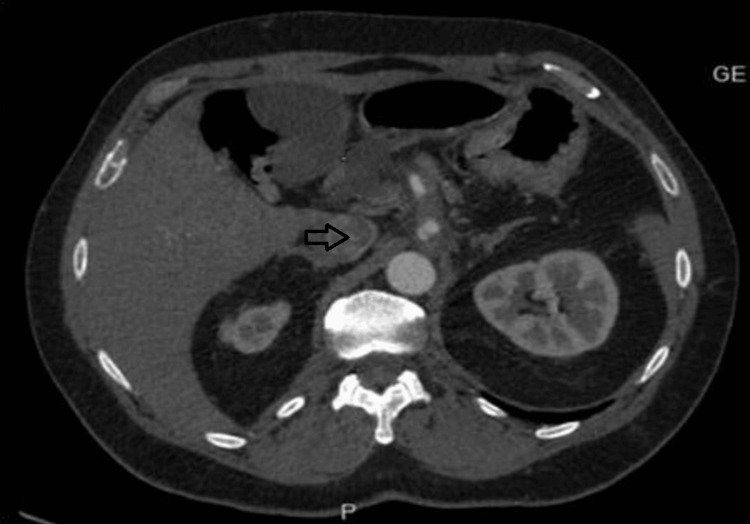
CT angiogram of the abdomen and pelvis showing a saccular pseudo-aneurysm (arrow)

A computerized tomography (CT) scan of the abdomen and pelvis without contrast was ordered because of the severity and duration of the patient's pain, as well as his significant past surgical history. The CT scan showed celiac artery enlargement with adjacent inflammatory stranding. There were other chronic findings secondary to prior surgery. A CT angiogram of the abdomen and pelvis was performed, which showed a 10 x 5 mm saccular pseudoaneurysm of the posterior aspect of the celiac trunk with surrounding inflammatory changes (Figure 1).

The patient’s pain was 8/10 on arrival and 3/10 after intravenous opioid medication was administered. He was evaluated in the emergency department by the surgical service and was admitted to the hospital for surgical management of the celiac artery pseudoaneurysm. After admission, an initial attempt was made to repair the celiac artery aneurysm with an endovascular approach based on the approach's less invasive nature. With local and moderate sedation, ultrasound-guided percutaneous access was achieved through the right common femoral artery. Cannulation of the celiac artery was achieved, however, aneurysm repair was unsuccessful, as the patient was not able to tolerate the procedure due to discomfort. A second attempt was made with access through the left brachial artery under general anesthesia. The aneurysm repair was successful after the placement of the stent graft. Repeat angiogram showed successful exclusion of the aneurysm, with excellent perfusion to the arteries distal to the site of repair. The patient was successfully discharged two days later with outpatient follow-up.

## Discussion

From a histopathologic point of view, all VAAs, including CAAs, can be divided into true aneurysms (maintaining all components of the arterial wall) versus pseudoaneurysms (which do not have all components of the arterial wall). Pseudoaneurysms occur within the adventitial layer after an intimal injury and are unstable [5]. The most common cause of true aneurysms overall is atherosclerosis. Other causes include connective tissue diseases and fibromuscular dysplasia [3]. The most common causes of pseudoaneurysms include trauma, infections vasculitis, and inflammation [1]. Specific etiologies for CAA include infectious diseases, atherosclerosis, trauma, congenital conditions, such as median arcuate ligament syndrome, and iatrogenic causes.

In reference to patient presentations with CAA, most cases of CAA progress asymptomatically. The most common presenting symptom, as in the patient presented, is abdominal pain. CAAs can present as upper gastrointestinal hemorrhage [3]. The most common site of pain from a CAA is in the epigastric area [3]. Other locations of pain, such as the left lower quadrant, can occur. CAAs can be identified incidentally on CT scans [1]. Concerning indications for treatment, because of the overall rarity of VAAs, there is no overall consensus concerning their treatment [2]. A rupture is a clear indication for surgery in VAAs, including CAAs [2,4]. The risk of rupture is related to size. For aneurysms 15-22 mm in size, the risk of rupture is 5%. An aneurysm greater than 30 mm has a 50-70% risk of rupture [4]. A CAA greater than 25 mm in size is an indication for surgery [1,4].

Interventions include open surgical repair as well as endovascular techniques for stent implantation or embolization of the aneurysm [4]. Surgical repair can be performed using prosthetic grafts or autologous (usually saphenous) vein graphs [2]. The specific treatment is based on anatomic location, urgency in reference to ruptured or non-ruptured status, and the presence of significant co-morbidities that might preclude open surgery [1]. Patients who are at high risk for open surgery may be candidates for a coil embolectomy [1]. The major challenge for endovascular techniques is when the CAA involves the origin of the celiac artery. This is because in such a scenario, there is no room for a seal zone for a stent graft and there may be no proximal space for coil embolectomy [1]. CAAs tend to be fusiform and are found in the distal third of the celiac artery [5]. Our patient had a saccular pseudoaneurysm.

VAAs diagnosed in pregnancy, including CAAs, may more likely need intervention, even if less than 25 mm in size, due to an increased risk of CAA rupture in pregnant patients [1]. There is an increased risk of rupture with inflammation. Significant abdominal pain with a CAA places the patient at increased risk of rupture [5]. Our patient had significant abdominal pain. As with any vascular repair surgery, revascularization of the peripheral vascular branches distal to the repair is critically important [6]. With celiac artery aneurysm repair, blood flow to organs supplied by the left gastric artery, common hepatic artery, and splenic artery should be verified to prevent ischemic damage [7]. Fortunately, anastomotic vessels between the celiac artery and the superior mesenteric artery provide collateral flow to most organs supplied directly by the celiac artery [8]. Overall, surgical treatment of VAAs, including CAAs, is said to have low perioperative morbidity and mortality, with a high long-term survival rate and a low incidence of long-term aneurysm-related complications [2].

## Conclusions

Celiac artery aneurysms can present to the emergency department with abdominal pain. Although rare, this type of visceral artery aneurysm carries a high mortality rate if ruptured and thus knowledge of this entity is of importance to the emergency physician. Surgical repair may be either with an endovascular approach or with open surgical repair of the aneurysm with prosthetic grafts. Endovascular approaches appear to be gaining increasing use.
